# Influence of C and N on Strain-Induced Martensite Formation in Fe-15Cr-7Mn-4Ni-0.5Si Austenitic Steel

**DOI:** 10.3390/ma14216502

**Published:** 2021-10-29

**Authors:** Caroline Quitzke, Qiuliang Huang, Horst Biermann, Olena Volkova, Marco Wendler

**Affiliations:** 1 Institute of Iron and Steel Technology, TU Bergakademie Freiberg, Leipziger Str. 34, 09599 Freiberg, Germany; olena.volkova@iest.tu-freiberg.de (O.V.); marco.wendler@iest.tu-freiberg.de (M.W.); 2 Institute of Energy Process Engineering and Chemical Engineering, TU Bergakademie Freiberg, Fuchsmuehlenweg 9, 09599 Freiberg, Germany; qiuliang.huang@iec.tu-freiberg.de; 3 Institute of Materials Engineering, TU Bergakademie Freiberg, Gustav-Zeuner-Str. 5, 09599 Freiberg, Germany; biermann@ww.tu-freiberg.de

**Keywords:** austenitic stainless steels, interstitial elements, in situ magnetic measurement, strain-induced α′-martensite, TRIP/TWIP effect, dynamic strain aging

## Abstract

In this study, the effect of interstitial contents on the mechanical properties and strain-induced martensite formation in an austenitic stainless steel was investigated. The mechanical properties of solution annealed Fe-15Cr-7Mn-4Ni-0.5Si-(0.01-0.2)N-(0.01-0.2)C concentrations in weight percent stainless steels were studied using room temperature tensile tests. All three alloys used in the present study have a sum content of C + N of about 0.2 wt.%. To verify the influence of C and N on deformation behavior, microstructural investigations are performed using light optical microscopy, scanning electron microscopy, and magnetic and hardness measurements. Moreover, strain-induced α′-martensite nucleation was characterized by scanning electron microscope using EBSD. In the present alloy system, carbon provides a stronger austenite stabilizing effect than nitrogen. Hence, the smallest amount of strain-induced α′-martensite was formed in the steel alloyed with 0.2 wt.% C. It also exhibited the optimal mechanical properties, including the highest ultimate tensile strength (1114 MPa), uniform elongation (63%), and total elongation (68%). Moreover, the interstitial content influences the occurrence of dynamic strain aging (DSA), which was only observed in the steel alloyed with carbon. With increasing C content, the triggering strain for DSA decreases, which can be confirmed by in situ magnetic measurements during tensile testing.

## 1. Introduction

Austenitic stainless steels with transformation-induced plasticity (TRIP) and twinning-induced plasticity (TWIP) effects have experienced increasing interest in recent years thanks to their attractive strength–ductility combinations. Pronounced strain hardening is achieved when the austenitic parent phase undergoes a martensitic transformation during plastic deformation (TRIP effect) [1]. The main factors affecting the strain-induced martensite formation are the chemical composition of steel, the temperature, and the plastic strain [2]. Depending on the temperature und constitution of the steel, the formation of stress- and strain-induced martensite can occur under external loading [3]. Thus, stress-induced martensite formation is observed when the γ → α′ transformation occurs below the yield point of austenite. Strain-induced martensite formation, on the other hand, takes place when the triggering stress coincides with or exceeds the yield strength of the austenite. α′-martensite can form in single deformation bands, intersections of slip bands, interfaces of ε-martensite plates, deformation twins, and grain boundaries, which are also known to be nucleation sites [3,4,5,6].

A significant increase in strength is observed when austenitic stainless steels are alloyed with interstitial elements—such as carbon and nitrogen—especially at low temperatures [7]. Nitrogen delays the sensitization of austenitic grains, and thus intergranular corrosion (IC), by slowing carbide precipitation kinetics and growth at the grain boundaries. Hence, the nitrogen concentration at the grain boundaries increases even at low N contents owing to its microsegregation behavior [8]. Carbon, manganese, and chromium have a lower concentration of free electrons in austenite compared with N and support covalent bonds, increasing the formation of clusters in Fe-Cr-N-C steel. The combination of C and N promotes stronger ordering of Cr atoms and leads to a higher interstitial solubility of N and C in the austenite [9,10,11,12].

In FCC alloys, dynamic strain aging (DSA) can occur, and result in the localization of mechanical deformation in the Portevin–Le Châtelier (PLC) deformation bands [13]. DSA is typically based on the elastic interaction of sliding dislocations with diffusing alloying elements and is associated with the appearance of serrations in the stress–strain curve [14,15,16]. At room temperature, DSA cannot be explained by long-range diffusion of substitutional elements such as Cr, Ni, or Mn, because they are considered immobile in the austenite. In addition, the diffusivity of solute N and C in the austenite is very limited and their diffusion to dislocation cores is quite unlikely. For high Mn TWIP steels with 0.6 wt.% C, Lee et al. [17] proposed that immobile C-Mn point defects, also called complexes, interacting with stacking faults during plastic deformation are the reason for the occurrence of DSA. Such complexes may consist of an interstitial element and a substitutional element, as is the case with N that gathers around Cr, or with C that gathers around Mn [18]. In TWIP steels, C-vacancy and C-C complexes are also possible, but one C atom in an octahedral interstice and one Mn atom is the most likely complex configuration [10].

There is a high affinity of C and Mn atoms to form short-range clusters (SRCs) in Fe-Mn-C alloys. This implies that the C atom is located on the octahedral interstitial sites and the Mn atoms are located on the six closest sites (CMn_6_). When a stress is applied, the planar slip of dislocations destroys the short-range C-Mn clusters as the C atom reorients from the octahedral to the tetrahedral position. The change in the position of the C atom provides temporary disruption of the C-Mn cluster, causing the strength to drop [17,19]. The SRC can be restored by a short-range jump of the C atom from tetrahedral position to nearby octahedral position owing to the low activation energy for reorientation of the C-Mn complex [13,17,20]. The interaction between clusters and partial dislocations during planar slip is considered to be the cause of dynamic strain aging (DSA) at RT in C containing high Mn TWIP steels [13,17].

As already mentioned, there is also a high affinity of N and Cr atoms to form N-Cr complexes as short-range ordering (SRO). Typically, an N atom is located in the vicinity of six Cr atoms [21]. Karlsen et al. and Ehrnstén et al. could only detect DSA in AISI 316 L steel at temperatures of 288 °C and 400 °C, respectively, well above RT [21,22].

The observed serrations in stress–strain curves caused by DSA can be divided into five types. Type A are periodic serrations characterized by an abrupt increase in stress followed by a decrease, commonly below the general stress level of the stress–strain curve. It results from the nucleation of a deformation band (PLC band) at the peak stress and continuous propagation in the strain direction [23]. In contrast to type A, type B serrations formed in quick succession owing to the formation of narrow shear bands, which propagate discontinuously or not at all. Another shear band is produced when the next peak stress is reached in an adjacent area or at the same distance from the first. Type B serrations oscillate about a general load level [23]. Type C serrations are characterized by load drops always below the general load level and occur at higher temperatures and lower strain rates in contrast to type A and B [23]. Type D serrations exhibit plateaus that give the stress–strain curve the appearance of a staircase. This can be explained by the occurrence of shear bands that do not strain harden in front of the moving band. A similar type to type A is type E, which emerges from the type A serrations at higher strain values [24,25].

In the present study, the influence of N and C concentrations in the austenitic steel Fe-15Cr-7Mn-4Ni-0.5Si-(0.01-0.2)N-(0.01-0.2)C was examined. The effect of N and C alone as well as C + N on the mechanical properties, e.g. yield and tensile strength, deformability, strain hardening, and the formation of strain-induced α′-martensite, was investigated with tensile tests and magnetic saturation measurements (MSAT). In addition, the triggering stress for the onset of strain-induced martensite formation and the influence of short-range order are considered in more detail. Based on the in situ measurement, the effect of DSA on martensite formation can be correlated.

## 2. Materials and Methods

This research was carried out on TRIP/TWIP steels with the nominal chemical compositions Fe-15Cr-7Mn-4Ni-0.5Si-(0.01-0.2)N-(0.01-0.2)C (wt.%). The vacuum induction melting facility VIM 12 from ALD Vacuum Technologies (Hanau, Germany) was used to produce laboratory melts in a nitrogen atmosphere with a nitrogen partial pressure of 650 mbar. The molten steels were poured into a water-cooled copper mold at a temperature of 1560 °C. The chemical composition and the stacking fault energy (SFE) of the steels are given in Table 1.

The stacking fault energy was estimated with Equation (1) from Dai et al. [26]. Element concentrations were entered in weight percent.

γ_SF_ (mJ/m²) = 39 + 1.59Ni–1.34Mn + 0.06Mn^2^–1.75Cr + 0.01Cr² + 15.21Mo–5.59Si–60.69 (C + 1.2N)^0.5^ + 26.27(C + 1.2N)(Cr + Mn + Mo)^0.5^ + 0.61[Ni·(Cr + Mn)]^0.5^ (1)

**Figure 1:**
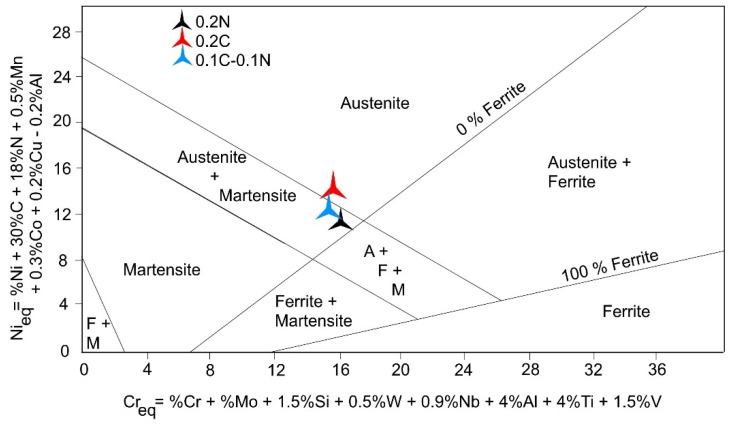
Positions of the examined steels in the Schaeffler diagram. The red and the black asterisks mark the steels with 0.2 wt.% C and 0.2 wt.% N, respectively. The steel containing 0.1 wt.% C and 0.1 wt.% N is indicated by a blue asterisk.

The SFE of austenite decreases with increasing carbon and decreasing nitrogen contents. Figure 1 presents the positions of the investigated steels in the Schaeffler diagram, which is very useful for predicting the phases present at RT [27,28]. According to the Schaeffler diagram, mainly, an austenitic microstructure is expected for the studied steels at room temperature.

After casting, the ingots were forged and hot rolled. The forged steels were subsequently austenitized at 1200 °C for 1 h and afterwards hot rolled into bars with a diameter of 12 mm. The bars were used for the production of tensile test specimens according to DIN 50125–B6x30. After fabrication, the specimens were solution annealed at 1150 °C for 30 min in a protective gas furnace with argon atmosphere and finally quenched in water.

Uniaxial tensile tests in crosshead-displacement control at an initial strain rate of 4 × 10^−4^ s^−1^ were carried out at RT using the tensile testing machine Inspekt200kN from Hegewald and Peschke GmbH (Nossen, Germany). In total, three specimens of each steel variant were tensile tested. The formation of strain-induced α′-martensite during tensile test was measured in situ. This method was proposed by Hauser et al. [29,30] based on a magnetic coil and data acquisition using LabView. The magnetic saturation analysis (MSAT) was used to quantify the ferromagnetic phase fraction with Lake Shore 480 model Fluxmeter from Metis Instruments & Equipment (Leuven, Belgium). Using thin slices with a thickness of 3.5 mm cut from the gauge section of the tensile specimens, the total volume fraction of strain-induced α′-martensite was used to calculate the in situ martensite evolution versus strain. In addition, further MSAT measurements were performed to gain knowledge about possible martensite formation in the thread of the specimens during the tensile test. 

The macro- and microhardness in HV10 and HV0.05, respectively, was measured with KB 30 S from Hegewald & Peschke GmbH (Nossen, Germany). For the microhardness, a step size of 0.05 mm was used between the hardness indentations. The microstructure was examined by means of light optical microscope (LOM) Axio Scope A1 from Zeiss (Oberkochen, Germany) and a field emission scanning electron microscope (FESEM). Using LOM, the average grain size was determined on five individual regions of specimens etched with HNO_3_. Prior to the microstructural examination with FESEM Ultra55 from Zeiss, the samples were vibration polished for 24 h to remove of preparation-induced martensite. With the aid of electron backscatter diffraction (EBSD), the distribution and orientation of martensite in the austenitic steel matrix was investigated. The EBSD measurements were performed using OIM XM4 system from AMETEK GmbH (Unterschleissheim, Germany) and evaluated with OIM Analysis^TM^ V8 software from AMETEK. Moreover, electron channeling contrast imaging (ECCI) was used to study the microstructure.

## 3. Results

### 3.1. In Situ Tensile Tests

**Figure 2:**
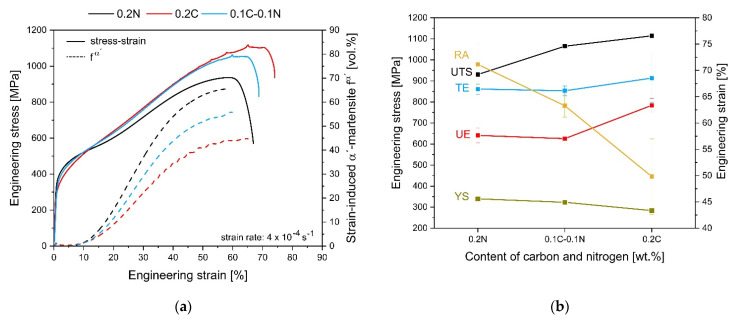
Tensile test results of the investigated steels: (**a**) engineering stress–strain curves and the evolution of strain-induced α′-martensite volume fraction $f^{\alpha \prime }$ at RT and (**b**) average mechanical properties as a function of the nitrogen and carbon content of the steels.

Figure 2 shows the tensile behavior and strain-induced α′-martensite evolution during tensile test at RT. In Figure 2a, the engineering stress–strain curves and the in situ strain-induced α′-martensite evolution are presented. The most important mechanical properties including ultimate tensile strength (UTS), yield strength (YS) R_p0,2_, uniform elongation (UE), total elongation (TE), and reduction of area (RA) depending on the content of carbon and nitrogen are shown in Figure 2b.

N steel has a typical S-shaped progression, common for high-alloy austenitic stainless steels exhibiting a α′-TRIP effect. In contrast, such a curve progression is less pronounced for the steels 0.2C and 0.1C-0.1N. Thus, the maximum achievable volume fraction of martensite ranged from 66 to 45 vol.% for the steels 0.2C and 0.2N, respectively, with the same degree of deformation. The steel with the same proportion of C and N shows mechanical properties, which were exactly between those of the steels 0.2C and 0.2N, respectively. Compared with the other steels, the 0.2C steel had the highest ultimate tensile strength and total elongation of 1114 MPa and 68%, respectively. Furthermore, it is shown that, with increasing carbon content and decreasing nitrogen content, both higher strength levels and deformations were achieved.

The 0.2C and 0.1C-0.1N steels show the dynamic strain ageing (DSA) phenomena at higher strains. The onset of the DSA effect was observed at 32% strain for the 0.2C steel and at 56% strain for the 0.1C-0.1N steel. In addition, dynamic strain aging also affected the in situ measured curve of strain-induced martensite formation. With the onset of DSA, the recorded martensite evolution curve showed plateaus and looked stair-like. The critical strain for DSA determined from this curve coincided with the strain value obtained from the tensile curves of the steels. The 0.2N steel exhibited no DSA (cf. Figure 2a). The periodic serrations in the stress–strain curves were classified as type A serrations [31,32].

As expected, the investigated steels showed an increase in martensite fraction with ongoing tensile deformation. The 0.2C steel exhibited the lowest strain-induced martensite formation compared with the other steels, but the highest strength. The highest strain-induced martensite fraction was measured in the 0.2N steel past to tensile test, which surprisingly had the lowest UTS of all steels.

### 3.2. Evaluation of the Influence of C and N on Short-Range Ordering and Clustering

In order to evaluate the interaction between the elements, the theoretical ordering index (TOI) can be used. The TOI is an empirical criterion for short-range order (SRO) and represents a measure of the proportion of C, N, Mn, and Cr atoms in different Fe-Cr-Mn steels. Owing to the strong affinity of Cr atoms and N atoms, this value can also be applied to N-Cr SRO. In principle, in FCC austenite lattice, C and Mn have a high affinity to generate clusters that consist of carbon atoms in octahedral interstitial sites and manganese atoms that occupy the six nearest neighbor sites (CMn_6_) [33].

In this study, the TOI value is calculated for C-Mn and N-Cr complexes and will be revisited in the following discussion on the strain hardening of the investigated steels, in addition to the influence of strain-induced martensite formation.

The TOI value is a semiempirical dimensionless parameter and was calculated with the following Equations (2) and (3) [34,35]. The abbreviation *x_i_* is the number of moles of the element *i*, *m_i_* is the mass of element *i*, and *M_i_* is the molar mass of element *i*. x_i1_ and x_i2_ represent the number of moles of interstitial and substitutional elements, respectively.

x_i_ (kg/kg·mol^−1^) = m_i_/M_i_, (2)

TOI (-) = x_i1_/x_i2_, (3)

For the interpretation of the TOI value, Saeed-Akbari et al. [34] presented the following. For TOI < 0.1—more than 10 Mn atoms are available for every C atom—the probability for the existence of CFe_6-x_Mn_x_ is high and the population of CMn_6_ clusters is very low. For TOI between 0.1 and 0.3 (10 Mn atoms for 3 C atoms), the number of C atoms increases and the population of CMn_6_ clusters increases too. In other words, the number of nearest neighbors around a C atom in the Mn-C complex affects the SRO. The driving force for SRO decreases as the number of Mn atoms in the next nearest neighboring sites of C atoms was reduced [34].

The TOI values for the C-Mn complex and Cr-N complex of the studied steels are presented in Table 2.

In summary, the TOI values of the studied steels indicates whether SRO or short-range clusters (SRCs) are present, which allows to estimate the expected influence on formability. With increasing C content and simultaneously decreasing N content, the tendency to form SRC increases, as is the case for the 0.2C steel. Consequently, the calculated TOI value for the Mn-C complex is 0.13 for the 0.2C steel, indicating that CMn_6_ clusters are preferred. Cr-N complexes in SRO are expected to be present in all studied steels, especially in the 0.2N steel, as the TOI value is less than 0.1. These agree very well with the investigations of Mosecker et al. [35] on Fe-14Cr-16Mn-0.3C-0.3N alloy.

### 3.3. Average Austenite Grain Size, Microhardness, and Microstructure

After solution annealing at 1150 °C for 30 min under argon atmosphere followed by water quenching to RT, the microstructure of all investigated steels consisted of austenite and δ-ferrite (<1 vol.%). The average grain size of the 0.2N steel was 45 µm after solution annealing. The average grain size of 0.2C steel and 0.1C-0.1N steel was 34 µm and 38 µm, respectively.

**Figure 3:**
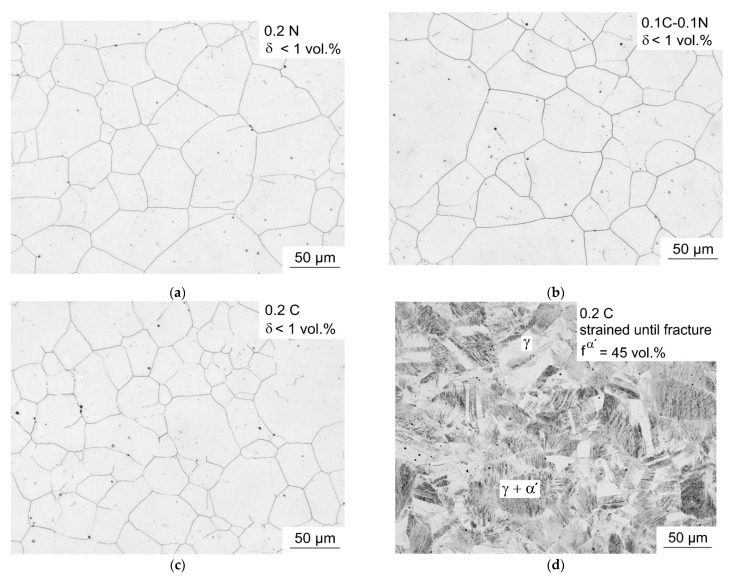
Light optical micrographs of the studied steels: austenite before tensile test (**a**) 0.2N, (**b**) 0.1C-0.1N, and (**c**) 0.2C; and (**d**) deformed microstructure of austenite and strain-induced α′-martensite of 0.2C steel after tensile test at RT until fracture. The tensile direction in (**d**) is normal to the plane of view.

Figure 3 shows the microstructure of the steels before tensile test (Figure 3a–c) and for the 0.2C steel after tensile test (Figure 3d). As the deformed microstructure of the other steels also consisted of austenite and strain-induced martensite, only the microstructure of 0.2C steel is exemplarily shown.

**Figure 4:**
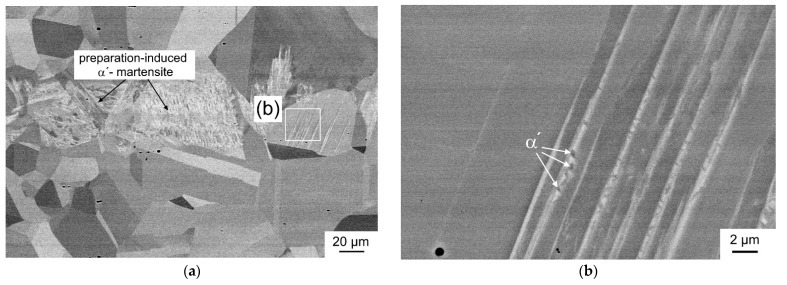
Microstructure of 0.2N steel in the threaded head after tensile test: (**a**) austenite grains with preparation-induced martensite and (**b**) detailed view of α′-martensite platelets nucleated in single deformation bands. The tensile direction is horizontal in the plane of view.

All investigated steels have a similar grain size before the tensile test and consisted of an almost fully austenitic microstructure with delta ferrite fractions below 1 vol.%. After RT tensile test, strain-induced α′-martensite was present in the deformed austenite (Figure 3d). The metastable austenitic steels were also examined in SEM. Figure 4 presents the microstructure of the 0.2N steel from a sample taken from the threaded head after tensile test at RT. In Figure 4a, preparation-induced martensite is clearly visible together with annealing twins in the austenite grains. The martensite was formed in a band-like region parallel to rolling direction across several austenitic grains. This observation is due to segregations formed during solidification of the ingot. As a result, the ability to form martensite is favored by cutting and grinding during metallographic specimen preparation in austenite depleted by main alloying elements. It was, therefore, not possible to completely prevent martensite formation during metallographic preparation [36]. As shown in Figure 4b, nucleation of preparation-induced α′-martensite is evident in straight deformation bands of austenite.

**Figure 5:**
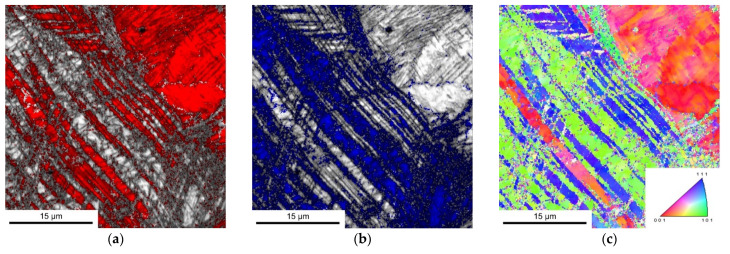
Microstructure of the 0.2C steel in the gauge section after tensile test at RT: (**a**) IQ + EBSD phase map of austenite (red), (**b**) IQ + EBSD phase map of strain-induced α′-martensite (blue), and (**c**) IPF map of austenite and α′-martensite. The tensile direction is normal to the plane of view.

Figure 5 presents the EBSD measurement results of a sample taken from the gauge section of 0.2C steel after tensile test at RT. The combined image quality (IQ) + phase map of austenite (FCC) and strain-induced α′-martensite (BCC) is shown separately. The maps allow to visualize the position of the phases in the deformed microstructure (Figure 5a,b). The martensite is formed inside the deformation bands of the austenite. The inverse pole figure (IPF) map of the microstructure (Figure 5c) clearly shows that a limited number of martensite orientation variants (green) were formed. This can be understood by variant selection during the strain-induced α′-martensite formation [37,38].

**Figure 6:**
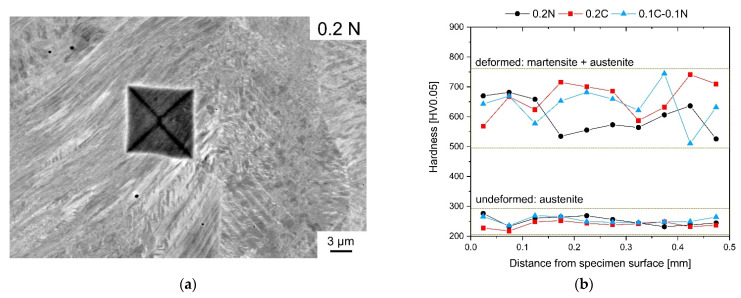
Microhardness profiles of the present steels after tensile test at RT: (**a**) SEM image showing a hardness indentation in the deformed microstructure of steel 0.2N and (**b**) hardness profiles taken from the initial undeformed and the deformed state after tensile test.

The Vickers hardness values of the steels with initial austenitic microstructure are in the range of 190 to 197 HV10. The microhardness profiles in HV0.05 were measured on cross-sectioned samples taken from the gauge length of deformed and undeformed round tensile specimens. Figure 6a presents a Vickers microhardness indentation in the deformed 0.2N steel.

Figure 6b presents the microhardness profiles for the investigated steels from the outer surface towards the center in the radial directions. The distance of the first indentation to the surface was 24 µm. Before the tensile test, the microhardness of austenite ranges from 232 to 276 HV0.05 and is very similar for all studied steels. The deformed microstructure consisting of strain-induced martensite and austenite possessed significantly higher microhardness values with respect to the initial state. The increased scatter of hardness values can be explained by the locally varying martensite and austenite volume fractions, which fluctuate between indentation points. In addition, an increasing C content seems to increase the microhardness, as demonstrated for the 0.2C steels in Figure 6b.

In summary, all investigated steels had an average grain size in a range of 34 µm to 45 µm. The initial microstructure after solution annealing was austenitic with a delta ferrite fraction smaller than 1 vol.%. After RT tensile test, the microstructure consisted of strain-induced α′-martensite and retained austenite.

## 4. Discussion

The investigated Fe-15Cr-7Mn-4Ni-0.5Si-(0.01-0.2)N-(0.01-0.2)C steels had different concentrations of C and N, which affected the tensile strength, strain-induced α′-martensite formation, and strain hardening. The stacking fault energy (SFE) was in the range of 17–20 mJ/m² at RT.

### 4.1. Strain Hardening and Triggering Stress of the α′-Martensite Formation

**Figure 7:**
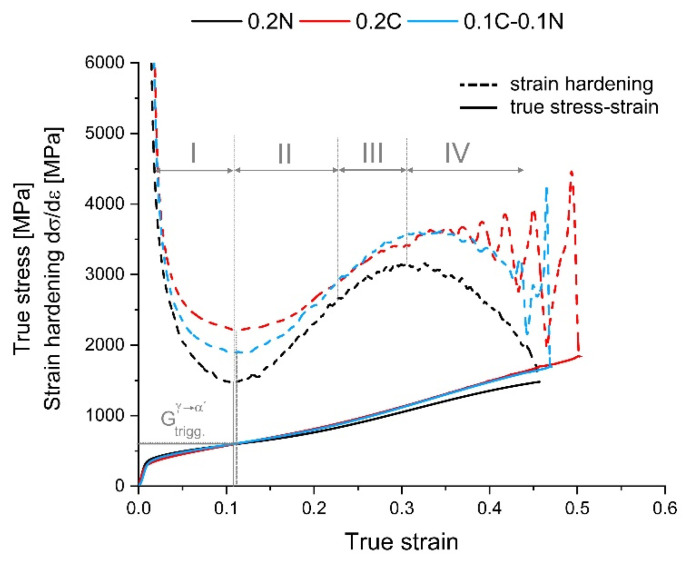
True stress–strain curves and strain hardening curves of the present steels at RT. The four stages of the strain hardening curve are exemplarily shown for the 0.2N steel.

Using the Considère criterion and the first derivative of the stress–strain curves, the strain hardening of the studied steels was calculated. Figure 7 summarizes the strain hardening curves of 0.2N, 0.2C, and 0.1C-0.1N steel. Generally, strain hardening of the investigated steels was mainly due to the increase in dislocation density and the interactions between dislocations and SROs in the early stage of tensile deformation. Once the triggering stress for strain-induced martensite formation was reached, the martensite fraction increased continuously with ongoing deformation. As a result, strain hardening was mainly affected by martensite formation [39,40,41].

The strain hardening curve of 0.2N steel has a lower minimum and reached nearly the same deformation than that of the 0.2C steel (Figure 7). The strain hardening curve can be divided into four stages mainly related to the strain-induced α′-martensite formation during tensile test [39,42,43]. In the first stage (stage I), the strain hardening decreased until a local minimum was reached. The minimum coincides with the first inflection point (IP) of the stress–strain curve and is often used to define the onset of strain-induced α′-martensite formation. However, from previous investigations into austenitic CrMnNi steels, it is known that a small strain-induced martensite fraction of 2–5 vol.% already exists in the minimum of the strain hardening curve [44,45,46]. Once the minimum in the strain hardening curve was exceeded, the curve increased progressively owing to the strong effect of strain-induced martensite on the strain hardening rate during ongoing tensile deformation (stage II). With start of stage III, the plastic deformation can no longer be fully accommodated by the plasticity of the austenite and the α′-martensite must be deformed. In stage IV, the strain hardening curve starts to decrease until necking occurs [47,48]. The true triggering stress and strain of the steels and the corresponding strain-induced α′-martensite volume fraction, related to the first IP of the stress–strain curve, are presented in Table 3.

The triggering stress and triggering strain slightly increase with the increasing content of carbon. The strain-induced α′-martensite fraction was in the range of 2.0 vol.% to 3.0 vol.%, and thus almost the same for all steels. In the 0.2N steel, martensite formation started slightly earlier, indicating the lowest austenite stability compared with the other steels.

The strain-induced α′-martensite formation is facilitated with an increasing nitrogen content and concurrently decreasing carbon content. The highest strength was obtained for the 0.2C steel. The true triggering stress for approximately 2–3 vol.% α′-martensite formation was in the range of 583 MPa to 619 MPa for all investigated steels. Despite an about 20 vol.% higher strain-induced α′-martensite volume fraction, a lower UTS was obtained for the steel 0.2N compared with the steel 0.2C.

It is noticeable that the 0.2N steel has a lower tensile strength, but the largest martensite volume fraction. This needs to be discussed in more detail. First in terms of martensite formation and then in terms of the elements C and N.

First, the α′-martensite formation in the studied steels can be explained as follows. Basically, there are different plasticity mechanisms in the studied steels. These are dislocation glide in the austenite, which could be planar or wavy, and the TRIP effect caused by the formation of strain-induced α′-martensite [49]. The SEM images (cf. Figure 5) show that the strain-induced martensite is formed in deformation bands. It is known that nucleation of strain-induced α′-martensite occurs by plastic deformation of metastable austenite [50,51]. According to the low SFE, partial Shockley dislocations dissociate and form extended stacking faults. These may accumulate in pronounced glide bands. The nucleation of strain-induced α′-martensite is preferred, e.g., in deformation bands, interfaces of ε-martensite plates, deformation twins, or grain boundaries [3,4,5]. The strain-induced α′-martensite is present in parallel deformation bands when only one set of deformation bands is dominant [51]. Owing to the formation of α′-martensite, the mean free path of dislocations is reduced with increasing strain [52]. The nucleation in single deformation bands indicates that the fraction of strain-induced α′-martensite is more related to the number of activated deformation bands and deformation band spacing than the number of deformation band intersections [53]. The nucleation occurs with respect to the dislocation motion on {111} lattice planes [54]. With increasing strain, the deformation bands with α′-martensite coalesce with neighboring deformation bands and form larger areas with strain-induced α′-martensite [55,56].

Second, all studied steels show an increase in ultimate tensile strength and uniform elongation with decreasing N and increasing C content. The investigations show a stronger stabilizing effect of carbon than that of nitrogen on the studied alloying system. The steel composition strongly affects the stability of austenite [2]. As a result, from the tensile tests, the strain-induced α′-martensite formation is less pronounced in the 0.2C steel, which proves that carbon is more effective in increasing the mechanical stability of austenite than nitrogen. The achieved experimental results agree very well with the investigations of Masumura et al. in Fe-18Cr-1Mn-8Ni-0.5Si-(0.001-0.1)N-(0.002-0.1)C steels [57]. Saenarjhan et al. studied Fe-15Cr-15Mn-4Ni-0.3Si-(0.01-0.3)N-(0.01-0.3)C steels with different N and C contents and concluded that N containing alloys exhibit a higher mechanical stability than C containing alloys [4]. In addition, short-range order (SRO) and short-range clustering (SRC) in solid solution were discussed to be the main cause of planar slip in FCC materials [58]. During strain hardening, planar slip occurs first, followed by the interaction of SROs and dislocations [34]. A Cr-N SRO in Fe-Ni-Cr-N steel at RT facilitates the formation of stacking faults and the evolution of planar dislocation slip, which should be most pronounced in 0.2N steel [59]. Owing to the presence of short-range ordered Cr-N complexes, dislocation movement requires additional force to pass local ordered zones, which contribute to strengthening in addition to strain-induced martensite formation. This results in increased solid solution strengthening [34,60,61,62]. Furthermore, the existence of SROs leads to a local increase in stress to activate dislocation glide. Consequently, the dislocation movement causes the destruction of the SRO and the following dislocation can glide more easily than on other planes [4]. In contrast, 0.2C steel has no SRO, but clusters and the explanation of 0.2N steel cannot be applied to 0.2C steel. The Mn-C cluster leads to a higher lattice resistance to dislocation glide, as passing through a partial dislocation leads to a change in the local position of interstitial atoms. A leading partial dislocation that passes a Mn-C cluster creates disorder, whereas a subsequent partial dislocation restores the FCC lattice. This disordering process results in a gradual reduction in stress required to move each of the following dislocations on the same glide plane. As a result, dislocation glide is restricted to a single glide plane and results in higher strain hardening [63].

### 4.2. Dynamic Strain Aging

The results of the tensile tests (cf. Figure 2a) show that dynamic strain aging (DSA) occurred with the increasing C content. In addition, DSA was also detected by in situ magnetic measurement. In Figure 2a and Figure 7, the serrated stress–strain curves and the fluctuations in the strain hardening rate are associated with DSA and can be described with the Portevin–Le Châtelier effect (PLC) [64,65]. The PLC bands form and propagate when the condition for DSA is met. This is the case when the temporarily arrested dislocations, which were fixed by point defects, are released simultaneously [66,67]. Furthermore, this is the cause of the serrations in the stress–strain curve.

DSA needs a critical strain ε_c_ to be reached [63,68]. The steel with the highest C content showed the most pronounced DSA behavior. For this steel, the critical strain for DSA was 32% and for the 0.1C-0.1N steel was 56%. In contrast, the steel with 0.2N showed no DSA at RT. For the studied steels, the critical strain increased with the decreasing C content. Thus, it can be shown that a higher C content shifts the critical strain to low strain values. A pronounced DSA can be explained by solute–dislocation interactions in the austenitic steel matrix [63]. Hence, the calculation of TOI values allows to estimate the preferred order of the interstitial elements C and N close to substitutional alloying elements. The calculated TOI value for the 0.2C steel given in Table 2 predicts short-range cluster (SRC) formation and short-range ordering (SRO) was estimated for the 0.2N steel. DSA leads to an increase in flow stress and strain hardening, but a decrease in post-uniform elongation and a reduction in area at fracture [63]. As shown in Figure 2b, the 0.2C steel possessed the lowest reduction in area after the tensile test due to DSA. The DSA observed in the C containing alloys can thus be explained by the strong interaction between C and Mn, leading to local ordering. Here, the C atoms are located on the octahedral interstitial sites and form clusters with the Mn as their nearest neighbors. This order leads to higher resistance of the lattice to dislocation slip, because the position of the C atom changes from the octahedral to the tetrahedral position during deformation [63]. The C atom, which was previously on the stacking fault plane, is thus moved to a higher energy tetrahedral position by the shear of the leading partial dislocation [17]. Then, the C atom re-orients by a single jump to a nearby octahedral position out of the stacking fault plane, and this process enhances planar glide [17]. The pinning of dislocations leads to a reduction in the number of mobile dislocations and an increase in flow stress as fewer dislocations have to move faster to accommodate the imposed strain rate [64]. The interaction between SRCs and partial dislocations during deformation is thus considered to be the cause of dynamic strain aging (DSA) at RT in the carbon containing austenitic steels. In contrast, for the nitrogen containing steels (with N-Cr as SRO), DSA occurs only at temperatures above 300 K for 316 LN steel because the activation energy for DSA is low. For a 316LN steel with 0.07 wt.% N, the activation energy for serrated flow is 111 kJ/mol (723 K) and 218 kJ/mol (923 K) by 0.22 wt.% N [69,70]. According to Ganesan et al. [69], the increase in activation energy with increasing nitrogen content may be attributed to the formation of N-Cr complexes. As the nitrogen content increases, more N-Cr complexes are formed, increasing the activation energy for the initiation of serrated flow [69,70]. Thus, a higher activation energy could be the reason for the absence of DSA in 0.2N steel at RT. In the case of the 0.1C-0.1N steel, owing to the lower C content, the critical strain for DSA is shifted to higher strains [71]. The suppressive effect of N on DSA could be caused by the increase in the flow stress, causing an increase in the actual stress and, consequently, changes in the deformation response [72].

**Figure 8:**
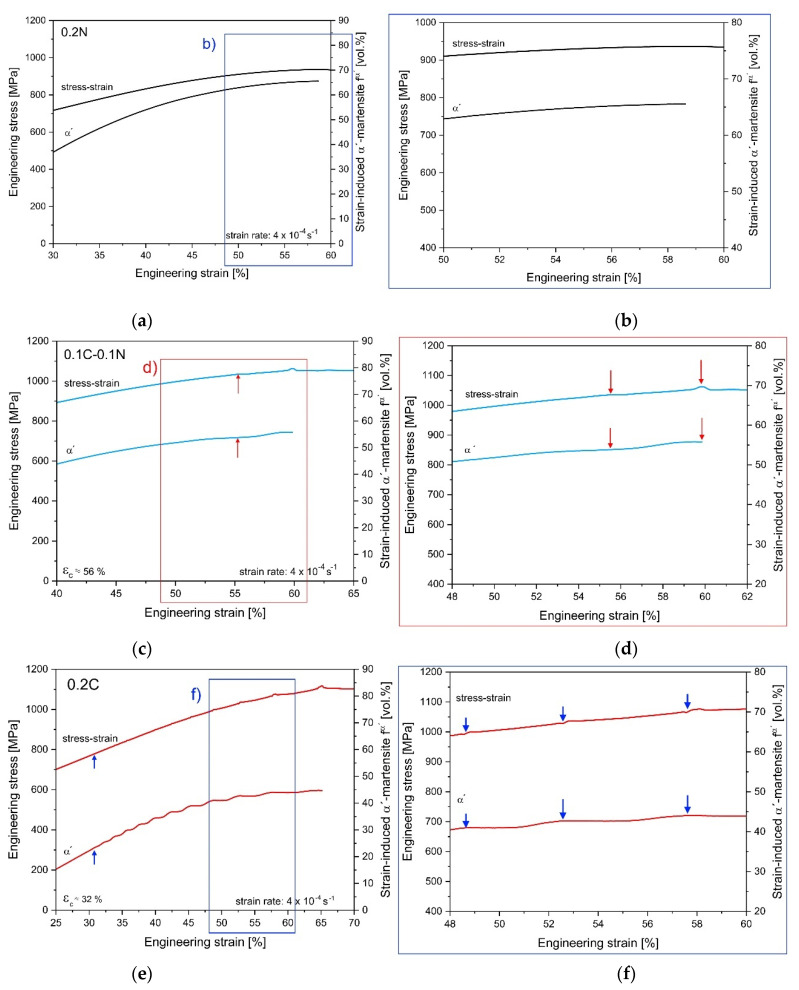
The stress–strain curve and strain-induced α′-martensite evolution with DSA phenomena at RT: (**a**,**b**) 0.2N steel without DSA, (**c**,**d**) 0.1C-0.1N steel with DSA from 56% strain, and (**e**,**f**) 0.2C steel with DSA from 32% strain. In (**c**,**e**), the red and blue arrows indicate the critical strain for the onset of serrated flow. (**b**,**d**,**f**) give the magnification of the areas framed in (**a**,**c**,**e**).

The serrations in the tensile curves of the studied 0.2C and 0.1C-0.1N steel shown in Figure 8 are classified to A type.

The red and blue arrows in Figure 8c,e, respectively, mark the onset of serrated flow. As already mentioned, the critical strain for DSA was reached earlier in 0.2C steel compared with 0.1C-0.1N steel. The high sensitivity of the in situ magnetic measurement for the determination of the martensite evolution allows to detect the dynamic strain aging very easily in the curve (cf. Figure 8e). Thus, there is a decrease in the martensite formation rate owing to the nucleation of a deformation band as the peak stress in the engineering stress–strain curve is reached.

As depicted in Figure 8a,b, the 0.2N steel shows the highest martensite formation capability and no DSA even at higher strains. This is in contrast to the investigations of Ogawa et al. on Fe-19Cr-8Ni-(0.05-0.14)C steel [73]. Ogawa et al. found that the γ→α′ transformation increased the local diffusivity of C owing to the change in crystal structure (FCC to BCC). They argued that, only in the presence of a high C concentration (0.14 mass% C) and a significant amount of strain-induced martensite (32 vol.%), is the condition for DSA fulfilled [73]. Ogawa et al. justified this by the formation of martensite, which allows increased local diffusivity of C in the plastically deformed region [73]. This is in agreement for the 0.2C steel, in which the first serration starts at 23 vol.% α′-martensite.

It is assumed that the Cr-N SRO influences the energy barrier from the austenite to martensite transformation [74]. In the 0.2N steel, the strain hardening is mainly caused by the martensite formation, which acts as an obstacle to dislocation movement. In contrast, strain hardening of the 0.2C steel is based not only on the formation of strain-induced martensite, but also on the dynamic strain aging in austenite caused by the C-Mn complexes, in which the change in the position of the C atom affects the dislocation motion [75]. Koyama et al. [76] also found no correlation between martensite formation and the formation of serrations, and concluded that the C atoms are triggers for the serrations. In addition, Koyama et al. [76] used an equation to calculate the critical strain for the onset of DSA as a function of carbon concentration and found that the critical strain for the onset of serrations decreases with an increase in carbon concentration. This result is in good agreement with our own observations.

## 5. Conclusions

The aim of this study was to investigate the mechanical properties and strain-induced α′-martensite formation behavior of Fe-15Cr-7Mn-4Ni-0.5Si-(0.01-0.2)N-(0.01-0.2)C austenitic stainless steels with different concentrations of interstitial elements. The strain-induced α′-martensite formation was studied with tensile tests and microscopic examinations (LOM, SEM). The main conclusions are as follows:

1. The 0.2C steel achieved the highest ultimate tensile strength (1114 MPa), uniform elongation (63%), and total elongation (68%) in contrast to the other studied steels, despite the lowest formation rate and total volume fraction of strain-induced α′-martensite.
2. The highest strain-induced α′-martensite fraction was obtained in steel 0.2N, which showed the lowest UTS and TE values.
3. The true triggering stress for the strain-induced α′-martensite formation slightly increased from 583 MPa to 619 MPa with the increasing carbon content. In 0.2N steel, martensite formation started slightly earlier, indicating the lower austenite stability compared with the other steels.
4. The different contents of C and N influence the potential of short-range ordering and clustering of atoms. The semi-empirical parameter, the TOI value, shows a favorable Cr-N short-range order in 0.2N steel, which affects SFE and dislocation glide. With increasing C content, the hardening increases and the strain-induced α′-martensite fraction decreases. For the 0.2C steel, the lowest strain-induced martensite formation (45 vol.%) and DSA phenomena were observed during tensile deformation.
5. The in situ magnetic measurement equipment is capable of detecting DSA phenomena in the steels. The serrations in the stress–strain curve caused by inhomogeneous plastic flow were also evident in the martensite formation curve.
6. DSA occurred in the 0.2C steel and 0.1C-0.1N steel. With the increasing C content, the critical strain for triggering DSA decreased.

## Figures and Tables

**Table 1:** Chemical composition of the investigated steels in wt.% and calculated SFEs at RT.

Alloy	C	N	Cr	Ni	Mn	Si	Fe + Others	SFE at RT [mJ/m²]
0.2N	0.01	0.22	15.0	4.0	7.1	0.5	bal.	20
0.1C-0.1N	0.10	0.12	14.7	4.1	6.9	0.5	bal.	18
0.2C	0.20	0.01	14.9	4.2	7.1	0.5	bal.	17

**Table 2:** Theoretical ordering index (TOI) of Fe-15Cr-7Mn-4Ni-0.5Si-(0.01-0.2)N-(0.01-0.2)C steels for the complexes C-Mn and N-Cr.

Alloy	TOI (-) C/Mn	TOI (-) N/Cr
0.2N	0.006	0.05
0.1C-0.1N	0.07	0.03
0.2C	0.13	0.002

**Table 3:** True triggering stress, triggering strain, and strain-induced α′-martensite fraction of the investigated steels in the first IP of the stress–strain curve.

Alloy	True Triggering Stress [MPa]	True Triggering Strain [–]	Strain-Induced α′-Martensite [vol.%]
0.2N	583	0.10	2.0
0.1C-0.1N	615	0.12	3.0
0.2C	619	0.12	3.0

## Data Availability

Data are contained within the article.
